# Eye movement training and gaze analysis for a patient with binocular diplopia after traumatic brain injury: a case report

**DOI:** 10.1186/s13256-023-04221-4

**Published:** 2023-12-18

**Authors:** Kaneharu Nakamura, Takeshi Fuchigami, Shu Morioka

**Affiliations:** 1 Department of Rehabilitation, Eishinkai Kishiwada Rehabilitation Hospital, 2-8‐10, Kanmatsucho, Kishiwada, Osaka 596-0827 Japan; 2 Stroke Rehabilitation Research Laboratory, Eishinkai Kishiwada Rehabilitation Hospital, Kishiwada, Osaka Japan; 3 Neurorehabilitation Research Center, Kio University, Kitakatsuragi-gun, Nara Japan

**Keywords:** Eye movement training, Gaze analysis, Binocular diplopia

## Abstract

**Background:**

Patients with traumatic brain injury often develop sequelae such as eye movement disorders, including diplopia. Eye movement training is effective in diplopia management. However, few longitudinal follow-up studies have been conducted from the subacute disease stage, owing to the complexity of methods for quantifying diplopia.

**Case presentation:**

The patient is a 30-year-old Japanese man who presented with diplopia and underwent eye movement training for approximately 4 weeks. The angle of diplopia, distance of abduction of the eye, gaze analysis, and self-assessment of diplopia using Holmes’ diplopia questionnaire were evaluated. The degree of diplopia increased from 12° to 40° on the right side. The distance of eye abduction increased from 10.4 to 12.8 mm. The self-assessment score improved from 76 to 12 points. Analysis of gaze transition revealed a reduction in the error between the target and gaze.

**Conclusion:**

Eye movement training was successful in ameliorating the symptoms of diplopia in the patient with binocular diplopia. Furthermore, for patients with diplopia symptoms, it was suggested that the oculomotor approach to eye movement training should not only focus on the direction of the diplopia but also on the direction opposite to the diplopia (the weak side).

**Supplementary Information:**

The online version contains supplementary material available at 10.1186/s13256-023-04221-4.

## Background

Approximately 90% of patients with traumatic brain injury develop ocular motility disorders, including binocular diplopia [1]. Binocular diplopia is a condition where one object is perceived as two objects located in different positions, caused by misalignment of the visual axis between the left and right eyeballs. It is due to dysfunction of the extraocular muscles [2], resulting from cranial nerve damage or damage to the nerves responsible for the control of eye movement [3]. The impairment of binocular function, including diplopia, significantly increases the likelihood of musculoskeletal injuries, fractures, or falls [4], which may have a deleterious effect on mobility, activities of daily living (ADLs), and recovery of quality of life; it also interferes with the effectiveness of treatment or rehabilitation [1, 5, 6].

Eye movement interventions regarding diplopia related convergence, pursuit, and saccades have been introduced as recovery therapies that entail direct retraining of impaired function [7, 8]; hence, its therapeutic effects on various eye movement disorders after traumatic brain injury have been demonstrated [9–14]. However, previous studies focused on vergence, myopia, and ocular motility disorders rather than diplopia symptoms. Moreover, there is a scarcity of studies on longitudinal follow-ups of patients from the subacute stage, because methods for quantifying diplopia are complex and require specialized equipment [15]. This case report describes a patient who presented with binocular diplopia after traumatic brain injury who underwent clinical evaluation of diplopia symptoms and gaze analysis.

## Case presentation

The patient is a 30-year-old Japanese man who fell down a hill while driving a tractor and was diagnosed with a depressed fracture of the right temporal bone as well as an epidural hematoma in the right temporal lobe using head computed tomography (CT). He underwent an emergency craniotomy on the same day for the evacuation of the hematoma due to decreased levels of consciousness and a tendency toward bilateral hematoma expansion. He was transferred to our hospital for rehabilitation after his general condition stabilized 40 days after admission. Prior to admission, the patient could independently perform ADLs and did not have ophthalmologic diseases such as strabismus or amblyopia. His chief complaint during admission to physical therapy evaluation was, “I want to reduce double vision.” He walked with the help of a walker and closed his right eye when performing movements due to diplopia. No motor paralysis or sensory impairment was observed during examination for physical function. Balance was measured using the Berg balance scale, which yielded a score of 54/56. Higher brain function was assessed using the mini-mental state examination-J, which yielded a score of 30. The results of parts A and B of the Trail Making Test were 42 seconds and 56 seconds, respectively. The patient was informed of the purpose, objectives, contents, and methods of the study both in writing and orally in accordance with the Declaration of Helsinki, and his written informed consent was obtained before the study was conducted.

### Diplopia and eye movement evaluation

**Fig. 1:**
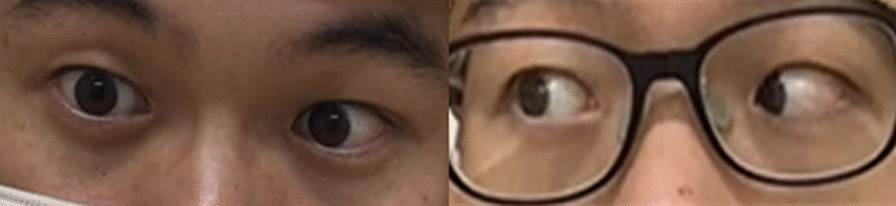
Eye movements to the right. Movements of right eye abduction were greater 2 weeks after intervention (right) than those before the intervention (left)

**Fig. 2:**
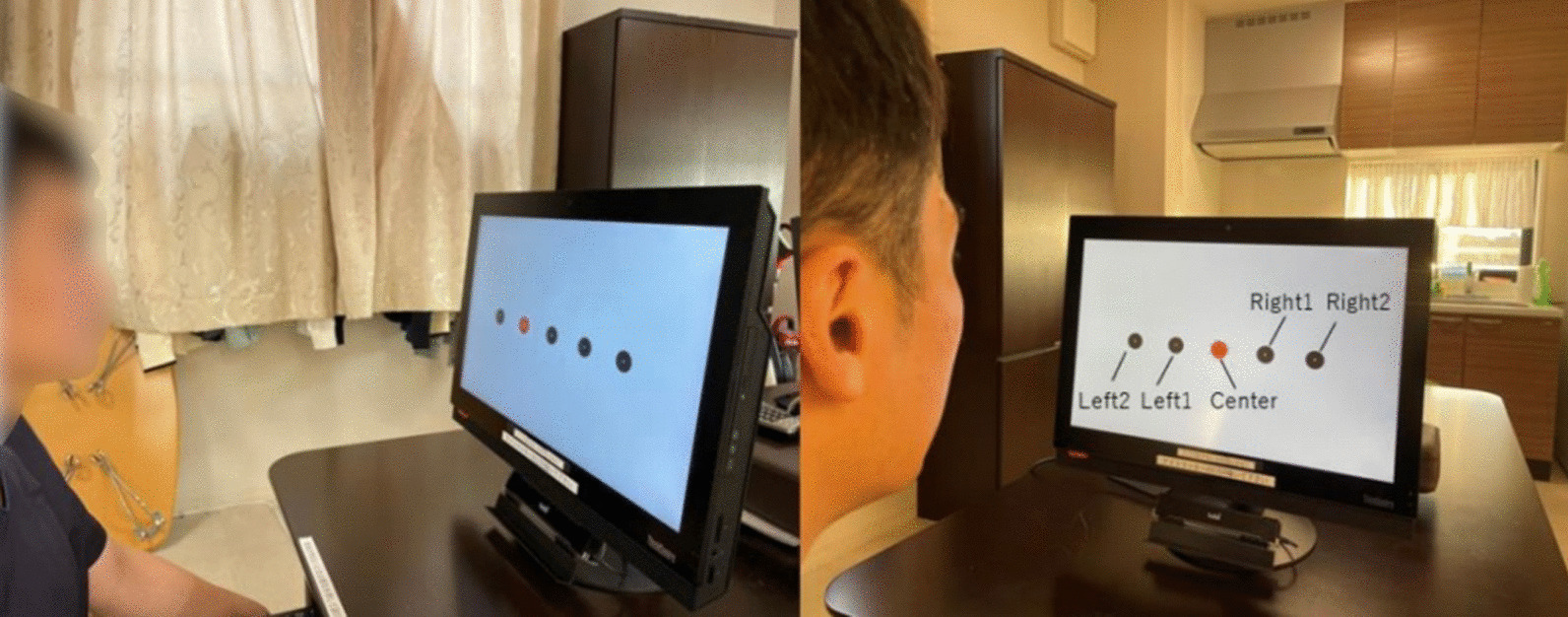
Scenario for the evaluation of eye movements. Eye movements require observation of five objects on the PC display that were detected using the eye tracker

Diplopia symptoms were observed at 12° from the primary position on the right side with the object in the midline, but losses or other defects of the visual field were not observed in any direction. Eye fatigue was observed after 3 minutes of eye movement training. The open-source image analysis software, ImageJ (https://imagej.net/downloads), was used to evaluate the amount (i.e., distance) of abduction of the right eye. The distance to the center of the pupil at maximum abduction of the right eye was 19.4 mm (Fig. 1). Holmes’ diplopia questionnaire (Table 1) was used for self-assessment of diplopia [16]. This questionnaire scores subjective diplopia symptoms in seven gaze positions, with higher scores indicating a greater symptomatic severity of diplopia. The patient’s score was 76/100 before the intervention. A computer display equipped with eye tracking hardware (Tobii TX60; TobiiInc., 1280 × 1024 pixels) was used to evaluate eye movements, which were recorded in response to each object displayed on the personal computer (PC) screen. We evaluated the gaze transition when the patient gazed at five objects (left 2, left 1, center, right 1, and right 2) presented horizontally on the display (Fig. 2). Assessments of gaze transition were conducted before the intervention, as well as 2 and 4 weeks after the intervention. For gaze analysis, we used the gaze coordinates at 0.8 seconds, approximately when the gaze reached the target after stimulus presentation. The average of the line-of-sight coordinates at that point, as well as the error between each target coordinate and the line-of-sight coordinate, were calculated and absolutized, followed by calculating the average magnitude of the error.

**Table 1:** Holmes’ diplopia question index

	Score if always	Score if sometimes	Score if never	Score
Straight ahead in distance	6	3	0	
Up	2	1	0	
Downstairs	4	2	0	
Right	4	2	0	
Left	4	2	0	
Reading	4	2	0	
Any position	1	1	0	
If “always, ” to all above, can you get rid of it?	−1			
			Total	

For each gaze position, a corresponding score of “always,” “sometimes,” or “never” is awarded. The total score is multiplied by four to obtain the final score; higher scores indicate greater subjective symptomatic severity of diplopia

### Intervention

**Fig. 3:**
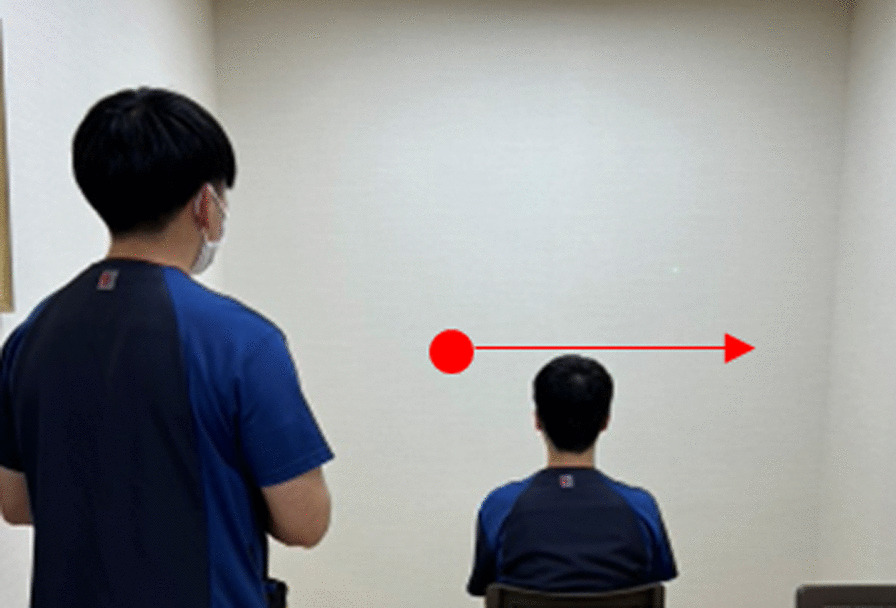
Representation of the tracking task using a laser pointer. The laser pointer moved slowly or quickly and the patient was instructed to follow it

**Fig. 4:**
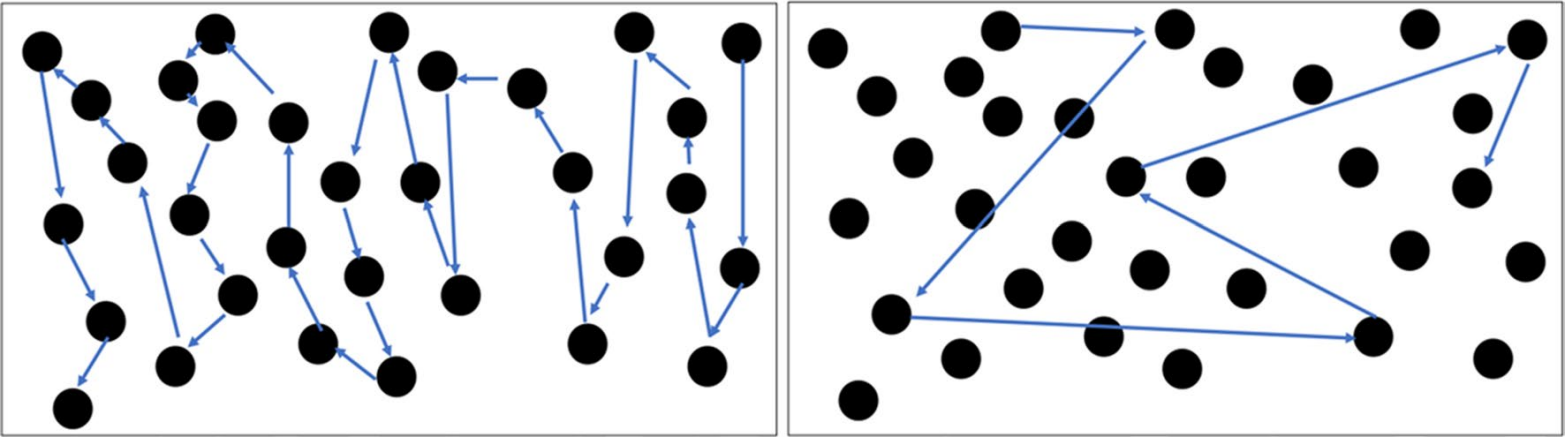
Task content. Sequential assignment (left) and random assignment (right)

Two 40-minute sessions of eye movement training were performed daily for 4 weeks. The training interventions included tasks such as following a laser pointer at slow and fast speeds as well as holding the laser pointer at the maximum eye position (Fig. 3). Moreover, 15 minutes of each 40-minute session were spent in the face-to-face sitting position using a touchscreen computer equipped with the rehabilitation tool @ATTENTION (manufactured by CREACT) to perform passive and random tasks (Fig. 4) in which the patient looked at the target. In the passive task, the objects were placed in an ordered fashion, and in the random task, one of the 35 objects placed randomly on the PC display flashed from black to red for about 5 seconds. The patient was instructed to gaze at the blinking object, with his own gazing point also being displayed in real-time and used as feedback. In addition to eye movement training, walking practice, ADL practice, and muscle strengthening training were performed daily for two 20-minute sessions.

### Results

**Fig. 5:**
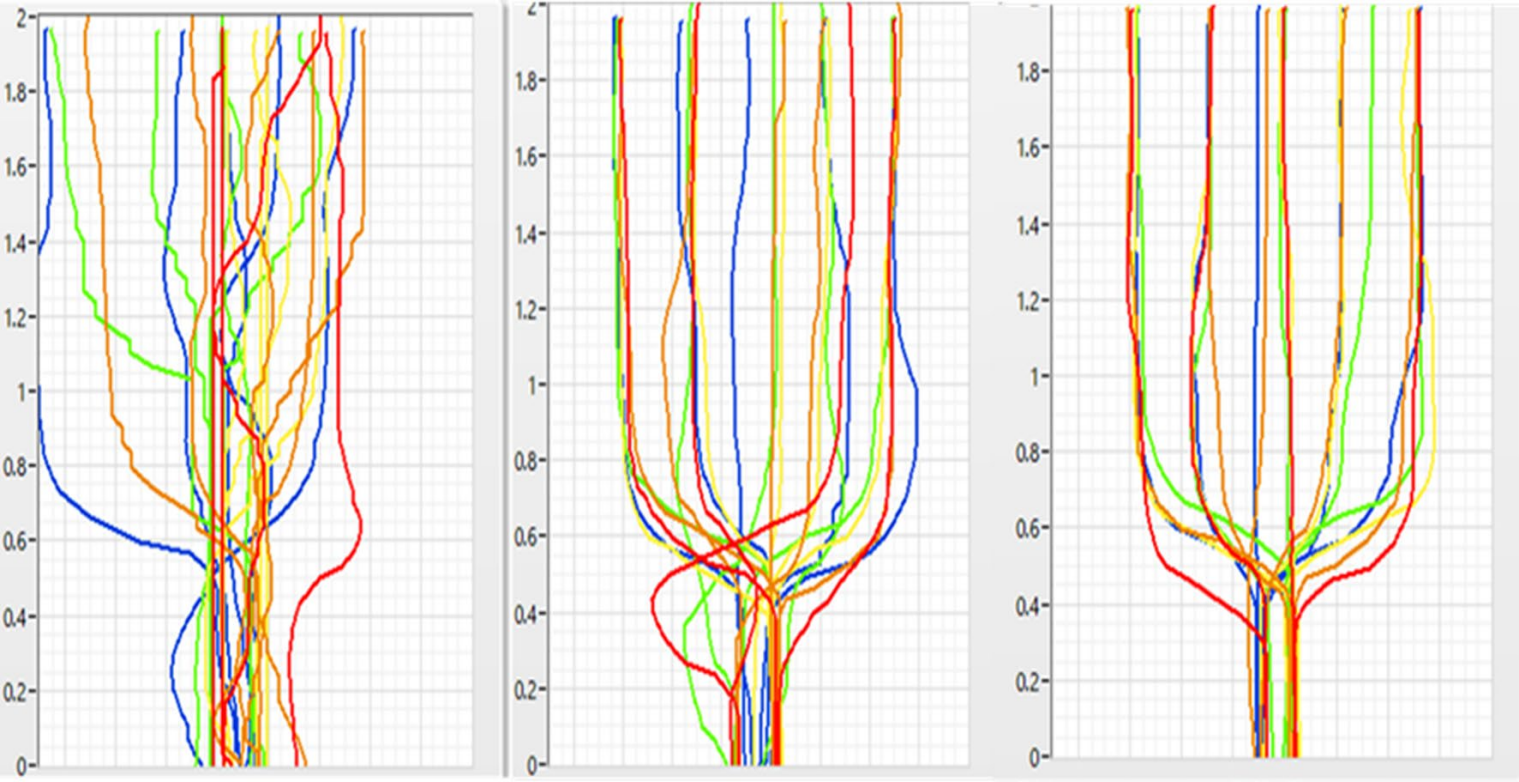
Results of gaze transition. Before the intervention (left), as well as 2 (center) and 4 weeks (right) after intervention. The variability in gaze decreased over the course of the intervention

**Fig. 6:**
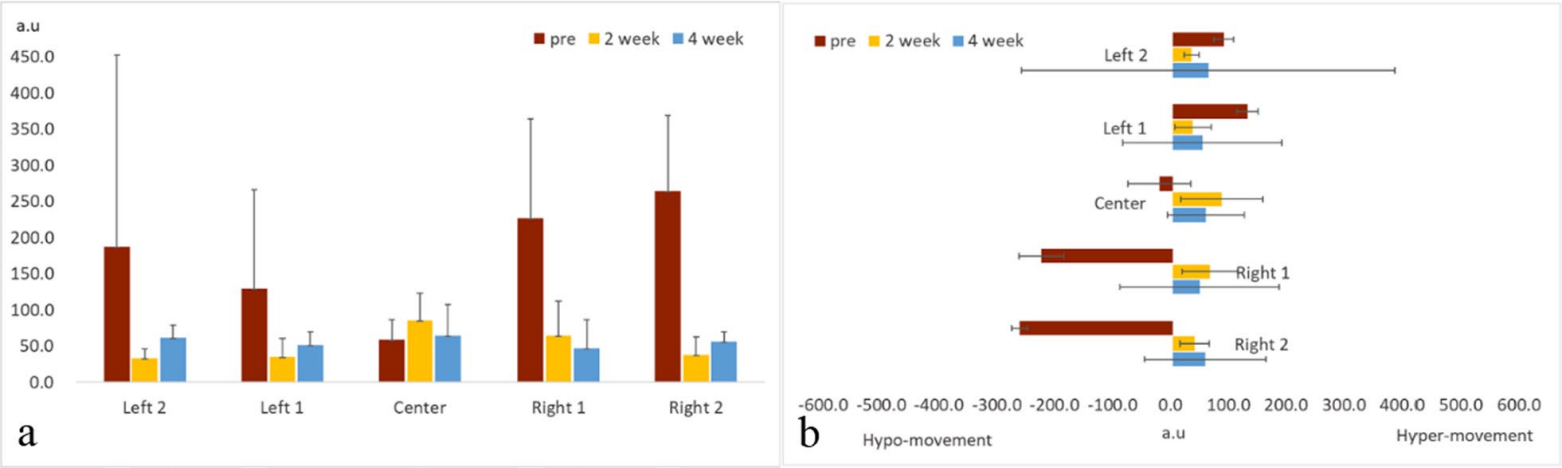
**A** Line-of-sight coordinates from each target (mean ± standard deviation). Before the intervention, an excessive leftward shift was observed when moving the gaze to left 2 and left 1, and the gaze did not reach right 1 and right 2. After 2 and 4 weeks of intervention, the excessive gaze shift to the left target was reduced and the deficient gaze shift to the right target was improved. **B** Errors between each target and line of sight (mean ± standard deviation). Before the intervention, errors with bilateral targets were larger. Errors with the targets left 2 and left 1 were larger than those with right 1 and right 2

Initially, the degree of diplopia was 12° to the right of the midline, which increased to 24° after 2 weeks of intervention, and to 40° after 4 weeks of intervention. The initial distance of abduction of the right eyeball was 19.4 mm, which improved to 23.6 mm after 2 weeks of intervention (Fig. 1) but remained unaltered after 4 weeks of intervention. The initial Holmes’ diplopia questionnaire score was 76 points, which improved to 26 and 12 points after 2 and 4 weeks of intervention, respectively (Table 2). Figure 5 depicts the trajectory of gaze transition before the intervention, as well as 2 and 4 weeks after the intervention. Gaze to target stabilization was visually confirmed at 2 (Fig. 5b) and 4 weeks (Fig. 5c) after the intervention compared with that before the intervention (Fig. 5a). Figure 6a shows the average gaze coordinates at each evaluation period minus the target coordinates, with positive values indicating excessive gaze transition and negative values indicating insufficient gaze transition. Before the intervention, the patient’s gaze shifted excessively to the left when shifting the gaze to the leftward targets (left 2, left 1), and did not reach the rightward targets (right 1, right 2). After 2 and 4 weeks of intervention, the excessive gaze transition to the target on the left side was reduced, along with improvements in deficient gaze transition to the right side. Figure 6b shows the magnitude of error between the target and the line of sight at each evaluation period. Before the intervention, the respective errors with the target on the left and right sides were significant. After 2 and 4 weeks of intervention, the errors between the left and right targets became smaller. However, errors with the left targets (left 2, left 1) were larger than those with the right targets (right 1, right 2).

**Table 2:** Eye Movement Assessment

	Before intervention	2 weeks	4 weeks
The maximum abduction of the right eyeball	19.4 mm	23.6 mm	23.6 mm
Diplopia symptoms	12°	24°	40°
Holmes’ diplopia question index	76	26	12

## Discussion and conclusion

In the present case, the distance of abduction of the right eye increased, and the degree of diplopia as well as the Holmes' diplopia questionnaire score, which were used to evaluate diplopia symptoms, also improved 4 weeks after eye movement training in the patient with binocular diplopia. Furthermore, the evaluation also revealed improvements in the distance and accuracy of gaze transition. The approaches used in case reports of previous studies include tracking eye movement training, saccade training, and convergence training, but they do not specify which direction the eye movement task is to be performed [9, 17]. Cochran also states that direct training and repetitive training to the injured area are effective in the rehabilitation intervention of ocular disorders [6].

In the present case, we visualized the gaze transition in the gaze analysis using @ATTENTION and calculated the errors from the target and the mean value of the gaze coordinates. We found that there were more errors from the target on the right side with symptoms of diplopia and on the left side without symptoms of diplopia. Bilateral eye movement training enabled the patient to maintain fixation on the fixation point and eye position, and improved the joint motion of the left and right eyes, leading to an improvement in the angle at which diplopia appeared.

These results indicate oculomotor learning and suggest functional recovery from oculomotor deficits, akin to previous studies [12–14]. Errors in target coordinates and gaze shifts were observed not only on the right side where diplopia symptoms were present, but also on the left side; thus, we suggest the need to involve the left side in rehabilitation practices, even if diplopia symptoms are not present on this side.

Eye movement training was performed with eye movement tracking tasks to both the left and right sides, but the patient had particularly strong complaints of diplopia symptoms and performed many eye movement transition tasks to the right side, where eye abduction movements were less frequent. A gaze shift task was then administered to the left side. Studies on rehabilitation for oculomotor disorders reported improved accuracy of eye movements and fixation after the intervention [17]. In the present case, gaze accuracy also improved, as evidenced by gaze transition evaluation. Eye tracking hardware was used as part of the target search task in this case, and its ability to provide real-time feedback about gaze transition may have contributed to its effectiveness. The patient’s symptoms of diplopia were ameliorated with an increase in the distance of abduction of the right eye.

The external ocular muscles responsible for eye movements sense stretch and contraction of the external ocular muscles themselves when the eye performs a purposeful movement and transmits this information to the visual center by coordinating eye movements and position. Without this information, the equilibrium between both eyes is disrupted, resulting in ocular positional abnormalities and diplopia [18]. This patient did not show signs of diplopia when gazing from the midline to the left side, resulting in monocular vision and eye movements. Therefore, we believe that the binocular diplopia in this patient was caused by a right external rectus muscle disorder, which was alleviated by the improvement in muscle function. In this case, there was no limitation of adduction in the right eye, but the patient had diplopia symptoms due to the lack of coordinated movement between the left and right eyes. This suggests the need to include the left side of the eye in diplopia treatment strategies, where diplopia is not present.

Before the intervention, the patient complained of a strong sense of anxiety during various activities because of diplopia, unless his right eye was closed, and he required supervision while performing activities in the hospital ward. The visual field and visual accuracy affect balance and motor skills [19], and alleviation in diplopia symptoms reportedly improves the functional independence measure and walking speed [20]. In the present case, the degree of independence experienced by the patient while performing ADLs also improved with reduction in the error of gaze transition and improvement in the degree of diplopia, which eliminated the need for monocular movements and expanded exploratory activities to the surroundings during movement. The evaluation of gaze transition revealed errors upon gazing at the target on the left and right sides where the left-sided diplopia had occurred, which persisted for 4 weeks after the intervention. During gazing, the coordination of the external ocular muscles, such as the external rectus and internal rectus muscles, facilitates the adjustment of gaze transition and fixation. The decrease in the coordination of the external rectus muscles involved in abduction movements is thought to have resulted in excessive gaze transition to the left side. Moreover, the intervention was performed with eye movement training to the right side, which might have resulted in residual errors when shifting the gaze to the left side. These results suggest the need for an approach that includes both the impaired and nonimpaired sides.

Our study has several strengths and limitations. The main strength of this study is that it was able to demonstrate improvements in diplopia symptoms despite the lack of research on rehabilitation for ocular motility disorders. In addition, we were able to visually show the gaze transition of patients with diplopia. These findings are useful for therapists of patients with diplopia as the intervention can be easily implemented in terms of time and frequency. A limitation of this study is that a single case experimental design was not introduced. Thus, the effectiveness of this intervention has not been verified. The applied behavioral analysis (ABA) B design was used for the study. Specifically, an ABAB design should be used to compare the results of each period.

In the present study, oculomotor training was performed on a patient with binocular diplopia, whose ocular motility disorder and diplopia symptoms were alleviated, and the patient could independently perform ADLs. Confirmation of the improvement in eye movement disorder and diplopia symptoms in conjunction with the evaluation of gaze transition clarified the need for training of both the affected and nonaffected sides.

### Supplementary Information

**Additional file 1**. CARE checklist.

## Data Availability

The datasets generated and/or analysed during the current study are not publicly available since they contain personally identifiable/ confidential medical information but are available from the corresponding author on reasonable request.

## Abbreviations

ADL: Activities of daily living
CT: Computed tomography
PC: Personal computer
