# Supplementary material for: Eye movement training and gaze analysis for a patient with binocular diplopia after traumatic brain injury: a case report
Source: J Med Case Rep. 2023 Dec 18;17:551. doi: 10.1186/s13256-023-04221-4 (PMC10726626; doi:10.1186/s13256-023-04221-4)
Supplement: Supplementary file 1 — Additional file 1. CARE checklist. [file 13256_2023_4221_MOESM1_ESM.pdf]

CARE case report guidelines

CARE Checklist of information to include when writing a case report

(cc) BY-NC-ND

Topic	Item	Checklist item description	Reported on Line	
Title	1	The diagnosis or intervention of primary focus followed by the words "case report"	P1	L1
Key Words	2	2 to 5 key words that identify diagnoses or interventions in this case report, including "case report"	P2	L18
Abstract (no references)	3a	Introduction: What is unique about this case and what does it add to the scientific literature?	P2	L13
	3b	Main symptoms and/or important clinical findings	P2	L6
	3c	The main diagnoses, therapeutic interventions, and outcomes	P2	L6
	3d	Conclusion—What is the main "take-away" lesson(s) from this case?	P2	L13
Introduction	4	One or two paragraphs summarizing why this case is unique (**may include references**)	P3	L1
Patient Information	5a	De-identified patient specific information.	P3	L24
	5b	Primary concerns and symptoms of the patient.	P4	L7
	5c	Medical, family, and psycho-social history including relevant genetic information	P4	L9
	5d	Relevant past interventions with outcomes	P1	L11
Clinical Findings	6	Describe significant physical examination (PE) and important clinical findings.	P4	L19
Timeline	7	Historical and current information from this episode of care organized as a timeline	P6	L6
Diagnostic Assessment	8a	Diagnostic testing (such as PE, laboratory testing, imaging, surveys).	P4	L19
	8b	Diagnostic challenges (such as access to testing, financial, or cultural)	P3	L23
	8c	Diagnosis (including other diagnoses considered)	P3	L23
	8d	Prognosis (such as staging in oncology) where applicable	P3	L23
Therapeutic Intervention	9a	Types of therapeutic intervention (such as pharmacologic, surgical, preventive, self-care)	P5	L18
	9b	Administration of therapeutic intervention (such as dosage, strength, duration)	P5	L18
	9c	Changes in therapeutic intervention (with rationale)	P5	L18
Follow-up and Outcomes	10a	Clinician and patient-assessed outcomes (if available)	P6	L6
	10b	Important follow-up diagnostic and other test results	P6	L6
	10c	Intervention adherence and tolerability (How was this assessed?)	P6	L6
	10d	Adverse and unanticipated events	P7	L17
Discussion	11a	A scientific discussion of the strengths AND limitations associated with this case report	P9	L20
	11b	Discussion of the relevant medical literature **with references**.	P7	L9
	11c	The scientific rationale for any conclusions (including assessment of possible causes)	P7	L14
	11d	The primary "take-away" lessons of this case report (without references) in a one paragraph conclusion	P10	L4
Patient Perspective	12	The patient should share their perspective in one to two paragraphs on the treatment(s) they received	P9	L6
Informed Consent	13	Did the patient give informed consent? Please provide if requested	Yes ☑	No ☐
